# Colorectal Cancer Surgical Pathology: Comparative Study in Lebanon Before and After the Economic Collapse

**DOI:** 10.1002/cnr2.70329

**Published:** 2025-09-09

**Authors:** Georges Ayoub, Rhea Khazen, Elie Berberi, Viviane Trak Smayra

**Affiliations:** ^1^ Faculty of Medicine Saint Joseph University of Beirut Beirut Lebanon; ^2^ Oncologic Pathology Department Dana Farber Cancer Institute Boston Massachusetts USA; ^3^ Anatomic‐Pathology Department Hotel Dieu de France Hospital, Saint Joseph University of Beirut Beirut Lebanon

**Keywords:** colorectal cancer, economic crisis, Lebanon, oncologic pathology, public health, surgical pathology

## Abstract

**Background and Objectives:**

Colorectal cancer (CRC) screening and early detection reduce mortality. Curative treatment is based on surgical resection, and pathological analysis plays a key role in management. In Lebanon, the impact of the COVID‐19 pandemic on healthcare has been compounded by an unprecedented socio‐economic crisis in 2020. This has deteriorated access to medical care and could have delayed CRC diagnosis and treatment. The study evaluates the impact of the economic collapse on CRC in Lebanon by comparing characteristics of patients operated on in 2020 and 2021 (post‐crisis period) to those operated on in 2018 and 2019 (pre‐crisis period).

**Methods:**

This retrospective study included patients who underwent surgical resection for colorectal adenocarcinoma at a tertiary care center in Lebanon between 01/01/2018 and 12/31/2021. Patients were divided into two groups according to the corresponding period, and the demographic, clinical, and pathological characteristics were compared.

**Results:**

Among the 169 patients included, 89 (53%) were operated on in the pre‐crisis period and 80 (47%) in the post‐crisis period. We reported that in the post‐crisis period: more patients presented with clinical complications requiring urgent admission (30% vs. 16%), more tumors were classified as T4b (10% vs. 2%), and more tumors presented lymphatic emboli (65% vs. 45%), perineural (51% vs. 22%) and perivascular invasion (40% vs. 19%) (*p* < 0.05).

**Conclusions:**

This study highlights the worsening of clinical and pathological indicators of poor prognosis by the current economic crisis, and this could potentially be translated into a reduction of survival.

## 1 Introduction

Colorectal cancer (CRC) is the third most diagnosed cancer worldwide and the second leading cause of cancer‐related death [1, 2]. In the Middle East and North Africa, Lebanon has the second highest incidence of CRC [3]. When diagnosed at an early stage, it is commonly treated by curative surgical resection [4, 5]. The surgical specimen is then analyzed in order to assign a pathologic TNM stage, which predicts the prognosis. The five‐year survival rate is estimated to be 60% for all stages combined, 90% for localized disease, 70% when there is regional extension, and 15% for metastatic CRC [2]. The development of invasive CRC commonly starts with the formation of a dysplastic adenoma [6]. Screening tests for CRC reduce the incidence of the disease, increase the number of cases diagnosed at an early stage, and reduce the mortality associated with CRC [7].

The COVID‐19 pandemic was responsible for a significant decrease in screening and detection of CRC, a trend that was maintained even after the removal of the restrictions [8, 9]. These studies also showed that the delay in diagnosis of CRC was responsible for an increase in emergency presentation of patients and a delay in management [10, 11]. Fewer cases were diagnosed at an early stage, compared to the pre‐pandemic period [12, 13].

In Lebanon, an unprecedented socio‐economic crisis, exacerbated by the explosion of the port of Beirut in August 2020, was added to the sanitarian effect of the COVID‐19 pandemic [14, 15]. These events resulted in a significant decrease in access to health care systems [16, 17]. This might have led to more emergency presentations of late‐stage diseases. There have been concerning reports on the impact of the crisis on the presentation and complicated clinical course of patients with this common disease during the pandemic [18]. This highlights the need for comprehensive, multi‐modal reporting and analysis of the colorectal cancer situation in Lebanon, including diagnosis, oncologic pathology, and the course of care.

This study aimed to comprehensively compare the demographic, clinical, and oncologic pathology characteristics of patients operated for CRC in 2018–2019 (before the crisis) and 2020–2021 (after the crisis) in order to evaluate the impact of the economic collapse in Lebanon on CRC presentation, prognosis, and management.

## 2 Materials and Methods

### 2.1 Population

This retrospective study evaluated all patients with adenocarcinoma of the colon or the rectum, admitted at Hotel‐Dieu de France (a tertiary medical care center in Lebanon), for surgical resection of CRC between the 1st of January 2018 and the 31st of December 2021.

The study included all the patients who were older than 18 years at the time of the surgery, had a confirmed adenocarcinoma of the colon or the rectum, underwent surgical resection of CRC between the 1st of January 2018 and the 31st of December 2021 regardless of the goal of the surgery (curative or palliative). Patients with recurrent disease following prior surgery were excluded from the study.

### 2.2 Demographic and Clinical Data

The demographic data included the biological sex and the date of birth. The clinical data included: date of the surgery, anatomic site of the tumor (right colon, left colon or rectum), presence of prior chemoradiation therapy, presence of metastasis at the time of diagnosis, and the clinical context of the patient's admission (pre‐scheduled or urgent surgery).

### 2.3 Pathology Data

The surgical pathology data include tumor site, tumor size, histologic subtype, grade, tumor extent, tumor budding score, presence of tumor deposits, presence of lymph vascular invasion, treatment response score if applicable, margin status, regional lymph node status, and mismatch repair proteins status. The pathologic stage was assigned according to the last edition of the TNM staging system of the American Joint Committee on Cancer (AJCC) and the International Union Against Cancer (UICC) [19].

### 2.4 Statistics

Patients were divided into two groups according to the date of the intervention, that is during the pre‐crisis period (2018–2019) or the post‐crisis period (2022–2021), and the variables collected were compared accordingly. Continuous variables were not normally distributed as per distribution evaluation with the Shapiro–Wilk test and hence are represented by the median value (Q1–Q3). The categorical variables were represented by their frequency (%). Statistical associations were evaluated using the Mann–Whitney *U* test for the continuous variable and the *χ* ^2^ test for categorical variables. A two‐sided *p* value of less than 0.05 was considered statistically significant. The statistical software JASP (Version 0.14.1) was used for analysis.

## 3 Results

### 3.1 General Characteristics

A total of 169 patients were included after 3 previously operated recurrent CRC cases have been excluded. The median age at the time of sampling was 66 (55–76) years. There was a male predominance with a sex ratio (M:F) of 3:2. Admission to surgery was pre‐scheduled for 131 (78%) patients and urgent for 38 (22%) patients. One third (*n* = 55) of the patients had rectal cancer, and the others had left (*n* = 46) and right (*n* = 68) colon cancer. Thirty‐three rectal cancer patients had received chemoradiation prior to the surgery (Table 1).

**TABLE 1:** General characteristics of patients (*N* = 169).

General characteristics	*N* (%) or median (Q1–Q3)
*N*	169
Age in years	66 (55–76)
Sex	
Male	100 (60)
Female	69 (40)
Colon cancer	114 (67)
Right colon	68 (60)
Left colon	46 (40)
Rectal cancer	55 (33)
Neoadjuvant treatment in rectal cancer	33 60)
Clinical context	
Pre‐scheduled admission	131 (78)
Urgent Admission	38 (22)

*Note:* Qualitative variables are represented in frequency (%) and Continuous variables are represented in median (Q1–Q3).

### 3.2 Pathology Characteristics

The majority of patients (*n* = 147) had Lieberkühnien NOS adenocarcinoma, and the histological subtypes includedmucinous adenocarcinoma (*n* = 17), ring‐cell adenocarcinoma (*n* = 3), micropapillary adenocarcinoma (*n* = 1), and undifferentiated (*n* = 1). Regarding local extension, tumors were classified as pTis, pT1, pT2, and pT3 in 4, 12, 22, and 75 patients, respectively. Tumors were classified as pT4 in 56 patients, of whom 46 were pT4a and 10 were pT4b. Half of the patients (*n* = 82) had lymph node metastases, 50 of which were classified as pN1 and 32 as pN2. Nine patients had pathologically confirmed distant metastases, and six had positive surgical margins. Half of the tumors were associated with lymphatic emboli, some with venous emboli (*n* = 56), perineural invasion (*n* = 61) and perivascular invasion (*n* = 49). The tumor budding score was low in 100 patients, moderate in 52 patients, and high in 17 patients. Subserous tumor deposits were observed in 34 patients. MMR status was classified as deficient (dMMR) in 25 patients (Table 2). Thirty‐three patients with rectal cancer received neoadjuvant treatment before surgical intervention (Table 3).

**TABLE 2:** Surgical pathology characteristics of the tumors (*N* = 169).

Pathology characteristics	*N* (%)
Adenocarcinoma	
Lieberkuhnian SAI	147 (87)
Mucinous	17 (10)
Ring‐cell	3 (2)
Micropapillary	1 (0.5)
Undifferentiated	1 (0.5)
pT	
pTis	4 (2)
pT1	12 (7)
pT2	22 (13)
pT3	75 (45)
pT4	56 (33)
pT4a	46 (82)
pT4b	10 (18)
pN+	82 (49)
pN1	50 (61)
pN1a	17 (34)
pN1b	22 (44)
pN1c	11 (22)
pN2	32 (39)
pN2a	18 (56)
pN2b	14 (44)
pM+	9 (5)
Stage	
pI	32 (19)
pII	54 (32)
pIIa	39 (72)
pIIb	15 (28)
pIIc	0 (0)
pIII	74 (44)
pIIIa	6 (8)
pIIIb	42 (57)
pIIIc	26 (35)
pIV	9 (5)
Positive margins	6 (4)
Grade	
Low	30 (18)
Moderate	121 (72)
High	18 (10)
Lymphatic emboli	92 (54)
Venous emboli	56 (33)
Perineural invasion	61 (36)
Perivascular invasion	49 (29)
Tumor deposits	34 (20)
Budding score	
Low	100 (59)
Moderate	52 (31)
High	17 (10)
dMMR	25 (15)

**TABLE 3:** Neoadjuvant rectal cancer treatment response (*N* = 33).

Tumor response score (*N* = 33)	*N* (%)
Tumor regression	
Complete or high response	3 (9)
Partial response	19 (58)
No or low response	11 (33)

### 3.3 Comparative Analysis: Pre‐Crisis and Post‐Crisis

**FIGURE 1:**
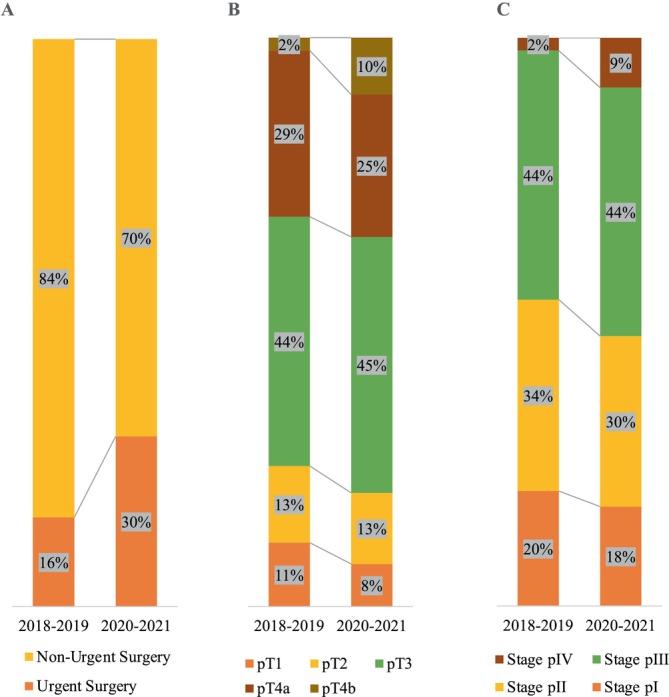
Overview of changes before and after the crisis (*N* = 169): (A) Clinical context at presentation, (B) tumor local extension, and (C) tumor staging.

**FIGURE 2:**
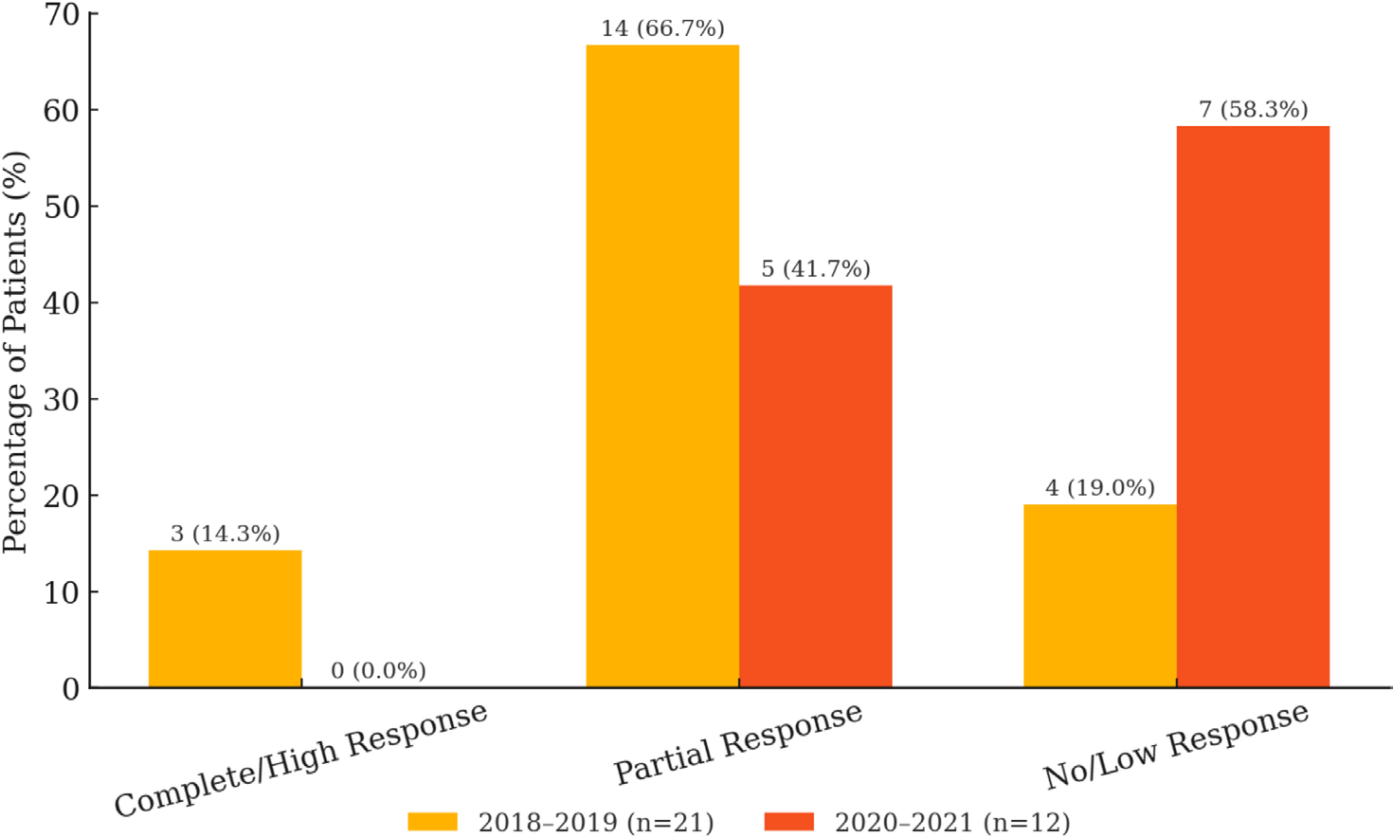
Neoadjuvant treatment response in patients with rectal cancer before and after the crisis (*n* = 33).

More patients were admitted via urgent surgical admission through the emergency department in the post‐crisis period (*n* = 24, 30%) compared to the pre‐crisis period (*n* = 14, 16%) (*p* = 0.03) . More tumors were classified as T4b in the post‐crisis (*n* = 8.10%) compared to the pre‐crisis period (*n* = 2.2%) (*p* = 0.03). Lymphatic emboli, perivascular, and perineural invasion were found more frequently during the post‐crisis period compared to the pre‐crisis period (*p* = 0.01). No statistically significant differences were found regarding age, sex ratio, tumor site, tumor size, histological subtypes, grade of differentiation, venous emboli, tumor budding, subserous tumor deposits, and lymph node metastases between the pre‐crisis period and the post‐crisis period (*p* > 0.05). A summary of the comparison of demographic, clinical, and pathological characteristics between the pre‐crisis period and the post‐crisis period can be found in Table 4 and Figure 1. As for the percentage of rectal cancer patients prescribed neoadjuvant treatment, 66% (21 out of 32) received neoadjuvant therapy in the pre‐crisis period, compared to 52% (12 out of 23) in the post‐crisis period (Figure 2). Among those who did not receive neoadjuvant treatment, 9% (1 out of 11) in the pre‐crisis group and 18% (2 out of 11) in the post‐crisis group were admitted urgently. As for the elective CRC cases that did not undergo neoadjuvant treatment, the median time from diagnosis to surgery was 21 days in the pre‐crisis period and 15 days in the post‐crisis period.

**TABLE 4:** Comparative analysis of the characteristics before and after the crisis (*N* = 169).

	Pre‐crisis	Post‐crisis	*p*
*N*	89	80	
Age in years	63 (54–73)	66 (58–77)	0.24
Sex			0.25
Male	49 (55)	51 (63)	
Female	40 (45)	29 (37)	
Localization			0.45
Right colon	36 (40)	32 (40)	
Left colon	21 (24)	25 (31)	
Rectum	32 (36)	23 (29)	
Urgent admission	14 (16)	24 (30)	**0.03**
Adenocarcinoma			0.23
Lieberkühnien SAI	78 (88)	69 (86)	
Mucineous	10 (11)	7 (9)	
Ring‐cells	0 (0)	3 (4)	
Micropapillary	1 (1)	0 (1)	
Undifferentiated	0 (1)	1 (1)	
High grade	10 (11)	8 (10)	0.13
pT4b	2 (2)	8 (10)	**0.03**
pN+	41 (46)	41 (51)	0.50
pM+	2 (2)	7 (9)	0.06
dMMR	13 (15)	12 (15)	0.94
Lymphatic emboli	40 (45)	52 (65)	**0.01**
Venous emboli	28 (31)	28 (35)	0.63
Perineural invasion	20 (22)	41 (51)	**0.01**
Perivascular invasion	17 (19)	32 (40)	**0.01**
Tumor deposits	13 (15)	21 (26)	0.06
Budding score			0.06
Low	60 (67)	40 (50)	
Moderate	21 (24)	31 (39)	
High	8 (9)	9 (11)	

*Note:* Qualitative variables are represented with *n* (%) and Continuous variables are represented with median (Q1–Q3). Bold values indicate statistical significance.

## 4 Discussion

In Lebanon, an unprecedented socio‐economic crisis, exacerbated by the explosion of the port of Beirut in August 2020, was added to the COVID‐19 pandemic that led to a reduction in CRC screening (8.9, 14, and 15). Our study was able to evaluate the impact of the economic collapse on CRC early surgical management by comparing demographic, clinical, and pathological data in the post‐crisis period to those of the pre‐crisis period. We herein shed light on the aggravation of poor prognosis indicators by the current socio‐economic crisis.

This study evaluated 169 patients operated for CRC in 2018‐2021. The main findings of this study reported that more patients had clinical complications requiring urgent admissions, more tumors were classified as T4b, and more tumors showed lymph‐vascular invasion in the post‐crisis period (2020–2021) compared to the pre‐crisis period (2018–2019).

This study reported that 30% of patients had clinical complications requiring urgent admission after 2019 compared to 16% in the pre‐crisis period. The inability of individuals and third‐party payers to cover health care costs may have led some patients to delay their clinical visits until they worsened enough to require an emergency room visit [20]. According to Western studies, delays in cancer screening during the pandemic also led to late diagnoses and a higher rate of patients being diagnosed in emergency settings [8, 10, 12, 21]. It should be noted that mortality and postoperative complications related to CRC are higher in patients admitted urgently than during elective admissions [22]. The slight decrease in the percentage of rectal cancer patients receiving neoadjuvant treatment during the pandemic compared to pre‐pandemic might suggest a delay in elective screening interventions. There was no increase in the time interval between diagnosis and surgery in our tertiary care center despite the challenges posed by the pandemic. Such consistency underscores the efficiency of oncology services strategy to prioritize surgical oncological procedures even amidst challenging circumstances potentially mitigating any adverse impacts of delayed resection after diagnosis on patient outcomes [23].

In our study, the tumors presented a more advanced locoregional extension since T4b was attributed to 10% of cases in 2020–2021 against 2% in 2018–2019. A T4b extension of CRC is defined when the tumor directly invades other nearby organs or structures. Direct invasion of adjacent organs has been shown by multivariate analyses to have a negative impact on prognosis [24]. After 2019, more tumors may have progressed to stage T4b due to delayed diagnosis or delayed treatment. The medical literature notes that a delay of more than 6 weeks in curative surgery for CRC leads to cancer progression [25]. Our finding is further supported by other studies which also reported a significantly higher rate of stage T4 in 2020 compared to 2019 due to the COVID19 pandemic [26].

Long before the socio‐economic crisis of 2020, it was already described in Lebanon that patients tended to present at advanced stages [27]. Indeed, in our study, about half of the patients presented with lymph node metastases, and this occurred in a similar way during the different periods.

It has been shown in several studies that the presence of lymphatic emboli, perineural or perivascular invasion constitutes an independent indicator of poor prognosis and a risk factor for hepatic or lymph node metastasis [28, 29, 30]. This study shows that the tumors of patients diagnosed during the post‐crisis period had more lymphatic, vascular, and perineural invasion. This could also be explained by a delay in the diagnosis of CRC during this period.

It is difficult to discern whether the late presentation of CRC in Lebanon is due solely to the pandemic or to economic deterioration, but it is highly likely that both factors contributed to our findings. Much effort is needed to make screening and early diagnosis of CRC accessible, especially during the economic difficulties that Lebanon is currently facing.

Some limitations in this study are worth to be considered. This study included patients who underwent a surgical resection of CRC and did not include patients with inoperable metastatic CRC. The proportion of patients with inoperable cancer may have increased accordingly. In addition, this study was carried out in a single surgical pathology department of a specialized tertiary care center in Lebanon, making it challenging to access the adjuvant treatment data of patients who may have had follow‐up in various dispersed medical oncology services. It would be valuable to confirm the effect of the crisis on long‐term survival with future prospective multicentered comprehensive clinical studies.

## 5 Conclusion

This study evaluated the impact of the economic collapse on the early surgical management of CRC patients by comparing demographic, clinical, and pathology data of the post‐crisis period to the pre‐crisis period. We herein shed light on the worsening of poor prognostic indicators by the current socio‐economic crisis as more patients had clinical complications requiring urgent admission, more tumors were classified as T4b, and more tumors showed lymph‐vascular invasion in the post‐crisis period compared to the pre‐crisis period. This could potentially be translated into a reduced survival of CRC in Lebanon.

## Author Contributions

**Georges Ayoub:** conceptualization, data collection, analysis, visualization, and writing. **Elie Berberi:** data collection and analysis. **Rhea Khazen:** analysis and writing. **Viviane Trak Smayra:** conceptualization, supervision, analysis, and writing.

## Ethics Statement

The study was evaluated by the local Ethics Committee of Saint Joseph University of Beirut, which raised no ethical objections and formally granted its approval in accordance with Good Clinical Practice as outlined in the Declaration of Helsinki and the international ethical guidelines for biomedical research involving human subjects issued by CIOMS and WHO.

## Conflicts of Interest

The authors declare no conflicts of interest.

## Data Availability

The data that support the findings of this study are available from the corresponding author upon request.
